# Two Patients with Fulminant* Clostridium difficile* Enteritis Who Had Not Undergone Total Colectomy: A Case Series and Review of the Literature

**DOI:** 10.1155/2015/957257

**Published:** 2015-11-22

**Authors:** Eliza W. Beal, Rosara Bass, Alan E. Harzman

**Affiliations:** ^1^Department of Surgery, The Ohio State University Wexner Medical Center, Columbus, OH 43210, USA; ^2^The Ohio State University College of Medicine, Columbus, OH 43210, USA; ^3^Division of Colorectal Surgery, Department of Surgery, The Ohio State University Wexner Medical Center, Columbus, OH 43210, USA

## Abstract

*Introduction*.* Clostridium difficile* is the most common cause of healthcare associated infectious diarrhea, and its most common clinical manifestation is pseudomembranous colitis. Small bowel enteritis is reported infrequently in the literature and typically occurs only in patients who have undergone ileal pouch anastomosis due to inflammatory bowel disease or total abdominal colectomy for other reasons.* Presentation of Cases*. We report here two cases in which patients developed small bowel* C. difficile* enteritis in the absence of these underlying conditions.* Discussion*. Neither patient had underlying inflammatory bowel disease and both had a significant amount of colon remaining.* Conclusion*. These two cases demonstrate that small bowel* C. difficile* enteritis should be included in the differential diagnosis of patients on antibiotic therapy who demonstrate signs and symptoms of worsening abdominal disease during their postoperative course, even if they lack the major predisposing factors of inflammatory bowel disease or history of total colectomy.

## 1. Introduction

*Clostridium difficile* is the most common cause of healthcare associated infectious diarrhea [1]. Symptoms and signs of severe* C. difficile* infection include watery, foul-smelling diarrhea, pseudomembranous colitis, marked peripheral leukocytosis, acute renal failure, and hypotension [2]. Exposure to antibiotics is the major risk factor for* C. difficile*. The incidence of* C. difficile* has increased significantly over the past 25 years with a concomitant increase in severity and mortality [2]. Intravenous metronidazole and oral vancomycin remain the treatments of choice for* C. difficile* colitis [2] and were long considered equivalent [3]. In a prospective, randomized, placebo-controlled trial it was demonstrated that the two agents had similar efficacy in mild infection, but that vancomycin had a significantly higher response rate in patients with severe infection (97% versus 76%, *P* = 0.02) [3]. Other studies have demonstrated similar results [4]. Other agents currently being evaluated include difimicin and tolevamer [2]. Small bowel enteritis is reported infrequently in the literature and is typically reported only in patients with inflammatory bowel disease (IBD) who have undergone ileal pouch anal anastomosis [5] or who have undergone total abdominal colectomy for other reasons [6]. The authors who have written on the topic seem to agree that early diagnosis is important in obtaining improved outcomes.

## 2. Presentation of Cases

### 2.1. Case 1

**Figure 1:**
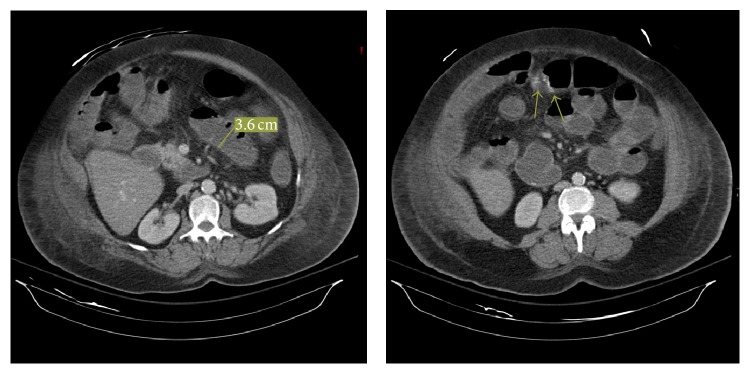
Computed tomography of the abdomen and pelvis with IV and PO contrast demonstrated diffuse dilation and fluid filling in the small bowel to the level of the ileocolic anastomosis out of proportion to the large bowel gas concerning for low-grade small bowel obstruction versus postoperative ileus.

A 63-year-old female presented for a right hemicolectomy and umbilical hernia repair for a large transverse colon polyp found on screening colonoscopy. She received 1.0 gram of ertapenem preoperatively. On postoperative day four she developed abdominal pain and distention, nausea, vomiting, and diarrhea. A nasogastric tube was placed which drained two liters of fluid. Computed tomography (Figure 1) demonstrated dilated small bowel loops with a small amount of gas in the colon consistent with an ileus. She developed hypotension, tachycardia, and reduced oxygen saturation and was transferred to the surgical intensive care unit (SICU) on mechanical ventilation and vasopressor support. She developed acute kidney failure. She was started on continuous venovenous hemodialysis to assist in metabolite and volume control and empiric broad spectrum antibiotics. On postoperative day five she was taken to the operating room for exploratory laparotomy. At the time of reexploration there was no acute intra-abdominal process noted, and her anastomosis was intact. She was transferred back to the SICU where she required increasing vasopressor support and continued mechanical ventilation. Flexible sigmoidoscopy was performed to the descending colon, showing no pseudomembrane formation nor other abnormalities. On postoperative day seven she had a cardiac arrest and expired despite attempted resuscitation. An autopsy was performed which demonstrated pseudomembranous enteritis throughout her small bowel with patchy transmural inflammation and hemorrhage and focal areas of necrosis. Opening of the small bowel demonstrated pseudomembranous exudates, which filled the lumen. The colon did not show pseudomembranes. The pathology of the resected mass demonstrated well-differentiated adenocarcinoma of the colon.

### 2.2. Case 2

A 63-year-old female presented with pelvic abscess and rectovaginal fistula several months after undergoing a low anterior resection at another institution complicated by intra-abdominal sepsis from dehiscence of her anastomosis. This had been treated with diverting loop ileostomy and closure of her abdominal fascia with biologic mesh. Since that time she had developed purulent drainage from her vagina and rectum. She had been started on per oral amoxicillin-clavulanate. She was taken to the operating room for low anterior resection with coloanal anastomosis, small bowel resection, primary repair of parastomal hernia with mesh, and drain placement. The patient was on broad spectrum antibiotics at the time of surgery. Specific preoperative antibiotics were not given. On postoperative day three she developed nausea and vomiting. On postoperative day four she developed hypotension. A nasogastric tube was placed with return of bilious output. She became hypotensive and was transferred to the surgical intensive care unit (SICU) for fluid resuscitation. She was afebrile, with moderate abdominal tenderness, reduced urine output, and elevated creatinine. The decision was made to proceed to the operating room. At the time of reoperation her anastomoses were intact, but the abdomen could not be closed. An ABThera Open Abdomen Negative Pressure Wound Therapy System was placed and she was returned to the SICU where she was started on intravenous metronidazole and oral vancomycin due to concern for small bowel* C. difficile* enteritis after pseudomembranes were noted on the ileostomy. A* C. difficile* toxin assay was positive at that time. Red rubber catheters were placed in both sides of her ileostomy and her rectum taking care not to disrupt her coloanal anastomosis. 500 mg of vancomycin was placed in each of these tubes and down her nasogastric tube every 6 hours.

Her hospital course was further complicated by ventricular tachycardia, heart failure intolerance of enteral feeds, cholestatic jaundice, need for reoperation for placement of absorbable mesh for closure of the abdominal cavity, leukocytosis, need for total parenteral nutrition (TPN), tracheostomy, development of enterocutaneous fistula, multiple intraabdominal abscesses, a repeat episode of* C. difficile* enteritis, atrial fibrillation with rapid ventricular rate, and a urinary tract infection. She remained in the hospital for two months.

At the time of discharge, her tracheostomy had been removed; she was tolerating snacks of thickened liquids, had a nasoenteric feeding tube in place, and was receiving TPN and intravenous metronidazole. A wound manager was being used to control her fistula output. The fistula subsequently closed, and she remains healthy but with a large ventral hernia.

## 3. Discussion and Review of the Literature

Clinical manifestations of severe* C. difficile* infection include watery, foul-smelling diarrhea, pseudomembranous colitis, marked peripheral leukocytosis, acute renal failure, and hypotension [2] with exposure to antibiotics as the major risk factor. Treatment for* C. difficile* infection includes intravenous metronidazole and oral vancomycin [2], which were long considered equivalent [3]. More recent studies have refuted this, suggesting that patients with severe* C. difficile* infection respond better to oral vancomycin [3, 4]. Other agents currently being investigated include difimicin and tolevamer [2]. Small bowel enteritis is reported infrequently in the literature and is typically reported only in patients with inflammatory bowel disease (IBD) who have undergone ileal pouch anal anastomosis [5] or who have undergone total abdominal colectomy for other reasons [6].

A literature review was performed. PubMed was queried using the terminology “*Clostridium difficile* enteritis.” There were 215 entries returned. These were examined for articles reporting cases of* C. difficile* enteritis. The references of these articles were also examined for additional cases. There were sixty-six cases reported in the literature between 1980 and 2013 (Table 1). Two cases were excluded due to the full-text article being in Dutch [7] and Spanish [8]. One case was excluded because full text of the article could not be located [9]. In total sixty-three cases were included in our analysis.

Of the 63 cases identified in the literature, thirty-one (49.2%) patients did not have a diagnosis of inflammatory bowel disease (IBD). Twenty-seven (42.8%) patients had ulcerative colitis and five (7.9%) patients had Crohn's disease. Fifty-five of the 63 (87.3%) patients received antibiotics in the time period preceding their development of* C. difficile* enteritis. Many of the patients received multiple antibiotics with 19 (30.2%) receiving cephalosporins, 12 (19.1%) receiving penicillin or penicillin derivatives, 13 (20.6%) receiving fluoroquinolone, 5 (7.9%) receiving aminoglycoside, 14 (22.2%) receiving metronidazole, 2 (3.2%) receiving carbapenem antibiotic, 4 (6.4%) receiving cotrimoxazole, 4 (6.4%) receiving vancomycin, and 1 (1.6%) receiving rifampin. Twenty-six (41%) of the patients had previously undergone a total proctocolectomy with ileal pouch anal anastomosis or total colectomy with end ileostomy for medically refractory ulcerative colitis. Thirty-two (50.8%) of the patients in this study had no remaining colon at the time they were diagnosed with* C. difficile* enteritis. Forty-four (69.8%) of the patients experienced resolution of their symptoms and 19 (30.2%) died [1, 10–47].

In keeping with our analysis, in a recent systematic review of the literature, Killeen et al. noted that the majority of reported cases occurred after colonic resection and that IBD in and of itself may predispose patients to* C. difficile* enteritis [48].

## 4. Conclusion

The patients presented here had several features in common. Both underwent surgery that altered their intestinal anatomy. In the first case this was a right hemicolectomy for colon cancer and in the second it was a redo low anterior resection. What is particularly interesting is that neither patient had underlying inflammatory bowel disease and both had a significant amount of colon remaining. While this is true of over half of the patients in the literature, the presence of pseudomembranes in the colon meant that, for one patient, this diagnosis was only accomplished during the autopsy. For the other a* C. difficile* toxin was isolated from the ileostomy output and pseudomembranes were visible on the stoma.

Many authors seem to agree that early diagnosis and treatment are keys to surviving* C. difficile* enteritis. Because this is a rare condition, especially in patients without underlying inflammatory bowel disease or history of total colectomy, the first patient was not diagnosed with* C. difficile* enteritis until autopsy. Our experience with this patient led to a higher index of suspicion in the second case, which allowed early identification of* C. difficile*. The timely treatment allowed the survival of this patient. These two cases demonstrate that small bowel* C. difficile* enteritis should be included in the differential diagnosis of patients on antibiotic therapy who demonstrate signs and symptoms of worsening abdominal disease during their postoperative course, even if they lack the major predisposing factors of inflammatory bowel disease or history of total colectomy.

## Figures and Tables

**Table 1:** Demographic features, risk factors, and outcomes of reviewed case reports. ^*∗*^IBD = inflammatory bowel disease.

*N* = 63 cases	Number	Percent/standard deviation
Crohn's disease	5	7.9%
Ulcerative colitis	27	42.9%
No IBD^*∗*^	31	49.2%
Median age	53.1	20.8
Received antibiotics		
Yes	55	87.3%
No	7	11.1%
Not reported	1	1.6%
Antibiotic type		
Cephalosporin	19	30.2%
Penicillin/penicillin derivatives	12	19.1%
Fluoroquinolone	13	20.6%
Aminoglycoside	5	7.9%
Metronidazole	14	22.2%
Carbapenem	2	3.2%
Vancomycin	4	6.4%
Rifampin	1	1.6%
Patients with total proctocolectomy with ileal pouch anal anastomosis	26	41.3%
Outcome		
Resolution	44	69.8%
Death	19	30.1%
